# OTULIN deficiency: focus on innate immune system impairment

**DOI:** 10.3389/fimmu.2024.1371564

**Published:** 2024-05-07

**Authors:** Bo Dou, Gang Jiang, Wang Peng, Chentao Liu

**Affiliations:** ^1^ Central South University, Xiangya Hospital, Pediatric Department, Changsha, Hunan, China; ^2^ Hunan Normal University, Hunan Provincial People's Hospital, Department of Respiratory Medicine, Changsha, Hunan, China

**Keywords:** OTULIN, deubiquitinase, genetic deficiency, autoinflammatory disorder, immunodeficient disorder

## Abstract

OTULIN deficiency is a complex disease characterized by a wide range of clinical manifestations, including skin rash, joint welling, lipodystrophy to pulmonary abscess, and sepsis shock. This disease is mechanistically linked to mutations in the *OTULIN* gene, resulting in an immune disorder that compromises the body’s ability to effectively combat pathogens and foreign stimuli. The *OTULIN* gene is responsible for encoding a deubiquitinating enzyme crucial for hydrolyzing Met1-poly Ub chains, and its dysfunction leads to dysregulated immune responses. Patients with OTULIN deficiency often exhibit an increase in monocytes, including neutrophils and macrophages, along with inflammatory clinical features. The onset of symptoms typically occurs at an early age. However, individuals with *OTULIN* haploinsufficiency are particularly susceptible to life-threatening staphylococcal infections. Currently, the most effective treatment for patients with *OTULIN* biallelic mutations involves the use of TNF-blocking agents, which target the dysregulated immune response. In conclusion, OTULIN deficiency presents a complex clinical picture with diverse manifestations, attributed to mutations in the *OTULIN* gene. Understanding the underlying mechanisms is crucial for developing targeted therapeutic interventions to address this challenging condition. Further research into the pathophysiology of OTULIN deficiency is essential for improving clinical management and outcomes for affected individuals.

## 1 Introduction

Immunological response, which is a body’s critical defense mechanism against pathogens and foreign substances, is tightly controlled at multiple levels including reversible modifications of various signaling molecules. Ubiquitination is an important post-translational modification that covalently attaches ubiquitin chains to target molecular proteins and either marks substrate proteins for proteasomal degradation or allows them to function as a scaffold to recruit other proteins and mediate downstream signaling (1–3). Linear ubiquitin chains are a unique type of ubiquitin chain, in which the ubiquitin molecules are linked together in a linear fashion through their N-terminal methionine and C-terminal glycine residues, and are generated by a multi-subunit E3 ligase complex called the linear ubiquitin chain assembly complex (LUBAC), which compromises three subunits: HOIL-1L (heme-oxidized iron-regulatory protein 2 ubiquitin ligase-1 like, also known as RBCK1), HOIP (HOIL-1L-interacting protein, also known as RNF31), and SHARPIN (SHANK-associated RH domain-interacting protein) (4, 5). Numerous publications reported functions of linear ubiquitin chains including regulating immune responses and inflammation, via NF-κB signaling in response to various stimuli, and preventing TNF-induced cell death (6–13). That disruption of LUBAC components leads to immunodeficient disorders and autoinflammatory disorders in humans (14), and embryonic lethality or inflammatory phenotype in mice (11, 12, 15), highlights the crucial role of LUBAC-mediated Met1 signaling in maintaining normal mammalian physiology. Ubiquitination is reversed by the deubiquitinating enzyme (DUB) via cleaving the ubiquitin molecules from their substrate (2). To date, almost 100 DUBs are identified in humans (2), but mainly A20, cylindromatosis (CYLD), and OTULIN have been characterized as key DUBs involved in the negative regulation of NF-κB activation and cell death (16). OTULIN, also known as Fam105B or Gumby, was recently identified in human and mice (17, 18), with exclusive specificity to hydrolyze Met1-poly Ub chains (4, 17–19). Herein, we briefly review the genetic and clinical features of OTULIN deficiency, focusing on immunological phenotypes. Finally, we summarize the state-of-the-art management options, shedding light on the current main research gaps.

## 2 OTULIN: structure, function, and mutations

**Figure 1:**
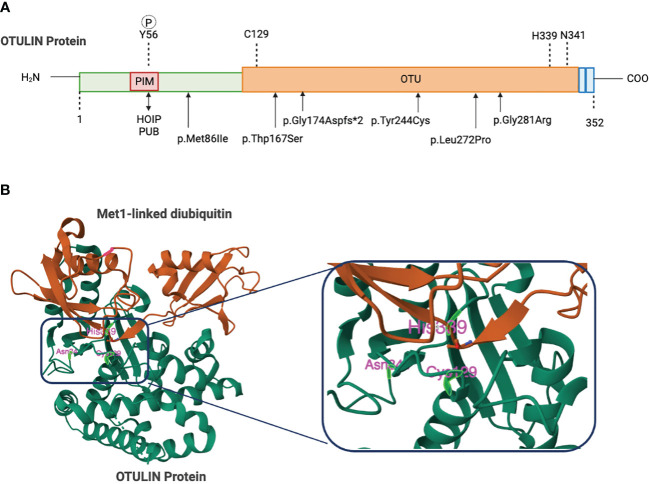
Domain organization of OTU deubiquitinates. **(A)** Schematic figure of the domain structures and important residues. The N-terminus of OTULIN contains the PUB-interaction motif (PIM) that mediates the interaction with the HOIP subunit of LUBAC. The OTU domain contains a catalytic triad consisting of Cys129, His339, and Asn342. The C-terminal OTULIN domain contains a PD2 binding motif (PDZbm) responsible for binding to the snx27. Gly174, Tyr244, Leu272, or Gly281 mutations in OTULIN catalytically inactivate OTULIN or reduce protein stability causing the severe inflammatory phenotype in ORAS patients. **(B)** Structure of OTULIN (green) bound to the Met1-linked Di-ubiquitin activating probe (brown) with a close-up view of the catalytic triad (Cys129, His339, and Asn341). Image created with Biorender.com.

The 352-amino acid protein OTULIN was initially discovered by Keusekotten et al. through a bioinformatics approach (17, 18, 20). This important deubiquitinating enzyme comprises a catalytic ovarian tumor (OTU) protease domain, an N-terminal interaction motif (PIM) involving peptide:N-glycanase (PNGase)/UBA-containing or UBX-containing proteins (PUB), and a C-terminal PSD95-Dlg1-ZO-1(PDZ) domain-binding motif (17, 18, 21, 22). OTU domain spans 80–345 amino acids, shares features of the papain-like family of cysteine peptidases, and employs a Cys129/His339/Asn341 catalytic triad (17, 20) (**Figure 1**). A striking function of OTULIN is its exclusive hydrolyzation to Met1-linked poly-Ub chains, without affecting other linkage types of poly-Ub chain (17, 18). This feature function originates from a highly conserved Ub binding site located in the OTU domain via a mechanism known as substrate-associated catalysis (17). Upon binding to Met1-linked poly Ub chains, the S1’ binding site in OTULIN orientates the Met1 residue of the proximal Ub toward the catalytic center, while the inter-Ub Lys residues remain remote from the catalytic center (17). However, binding of Ub chains with different linkages, but structurally similar Lys63-linked chains, would rotate the proximal Ub for several degrees and restrict proper binding to the S1’ binding site (17, 20). Ub substrates have also been demonstrated to directly contribute to OTULIN catalytic activity (17). In the absence of Ub, OTULIN Cys129, His399, and Asn341 adopt an inhibited catalytic triad conformation, and this auto-inhibition is released upon binding to Met1-linked proximal Ub (17). In addition, OTULIN selectively interacts with the LUBAC HOIP PUB domain via the N-terminal PIM, which covers Asp54–Met55–Tyr56–Arg57–Ala58 residues (21, 22) and serves as the sole site of interaction between OTULIN and HOIP, whereas the OTU domain’s presence is unnecessary for binding to HOIP (21, 22). Mutations in the OTULIN catalytic Cys129 do not affect the association between OTULIN and HOIP (15, 21).

Pathogens, tissue damage, and stress provoke host immune response and activate the downstream inflammatory signaling pathway. While immune receptors, such as TNFR1, NOD-like receptors (NLRs), CD40, toll-like receptors (TLRs), and the interleukin-1 receptor (IL-1R) instigate the assembly of receptor signaling complexes (RSCs), LUBAC recruitment via a Lys63 ubiquitin-dependent approach is a pivotal step in the course of these complex processes (5, 23–26). LUBAC recruitment facilitates Met1-linked ubiquitination of TNFR1, receptor-associated proteins, or pre-existing Lys63 polyubiquitin chains, and initiates downstream MAPK and NF-κB signaling by orchestrating the recruitment of the TAK1 and IKK complexes (24, 25, 27, 28). Numerous studies have demonstrated OTULIN’s role in counteracting LUBAC-mediated Met1 ubiquitination and subsequent NF-κB activation (17, 18, 29–33). However, deficiencies in OTULIN, overexpression of catalytic inactive OTULIN mutants, and OTULIN knockdown result in a remarkable increase in cellular Met1 Ub levels, leading to the accumulation of Met1-linked Ub on several pivotal proteins within inflammatory pathways, including TNFR1, receptor-interacting serine/threonine-protein kinase-1 (RIPK1), receptor-interacting serine/threonine-protein kinase-2 (RIPK2), NF-κB essential modulator (NEMO), and LUBAC (17, 23, 31–35). By contrast, overexpression of OTULIN leads to a reduction in TNFα-induced NF-κB activation and gene transcription (17). It is worth noting that unlike CYLD and A20, which exert their influence on NF-κB signaling through negative feedback mechanisms, OTULIN is not subject to transcriptional regulation by NF-κB (17, 18, 32, 36, 37).

The *OTULIN* gene is located on chromosome 5p15.2. To date, approximately 99 pathogenic or likely pathogenic germline variants are identified, affecting nearly 50 individuals (29–31, 38–40). Patients with variants in *OTULIN* gene and size of variants larger than 50 bps mainly present 5p- syndrome and are responsible for 72% of cases. Single genetic variants in *OTULIN* only account for 20%–30% of cases. Among these cases with single genetic mutation in *OTULIN*, missense mutations in OTULIN clustered within the OTU region underlie roughly 62.5% of cases, whereas null mutations (frameshift, nonsense, and splice site), located prior to or within the OTU region, represent approximately 37.5% of mutations (29–31, 39, 40). The impact of mutations in the *OTULIN* gene is quite variable, resulting in a range of clinical phenotypes. Loss-of-function mutations in the *OTULIN* gene often led to reduced levels of the OTULIN protein, which, in turn, contributes to the observed variability in clinical presentation. However, some mutations, such as OTULIN (L272P), are associated with only minimal degradation of the OTULIN protein (30, 31). Others, like OTULIN (G281R), result in almost complete loss of function (29). These findings underscore the complex and diverse effects of *OTULIN* gene mutations on protein expression and function, highlighting the need for further research to fully understanding the underlying mechanisms and potential therapeutic implication.

## 3 Clinical features: autoinflammation and immunodeficiency

### 3.1 Biallelic homozygous variants in OTULIN result in an early-onset autoinflammatory disease: OTULIN-related autoinflammatory syndrome

**Figure 2:**
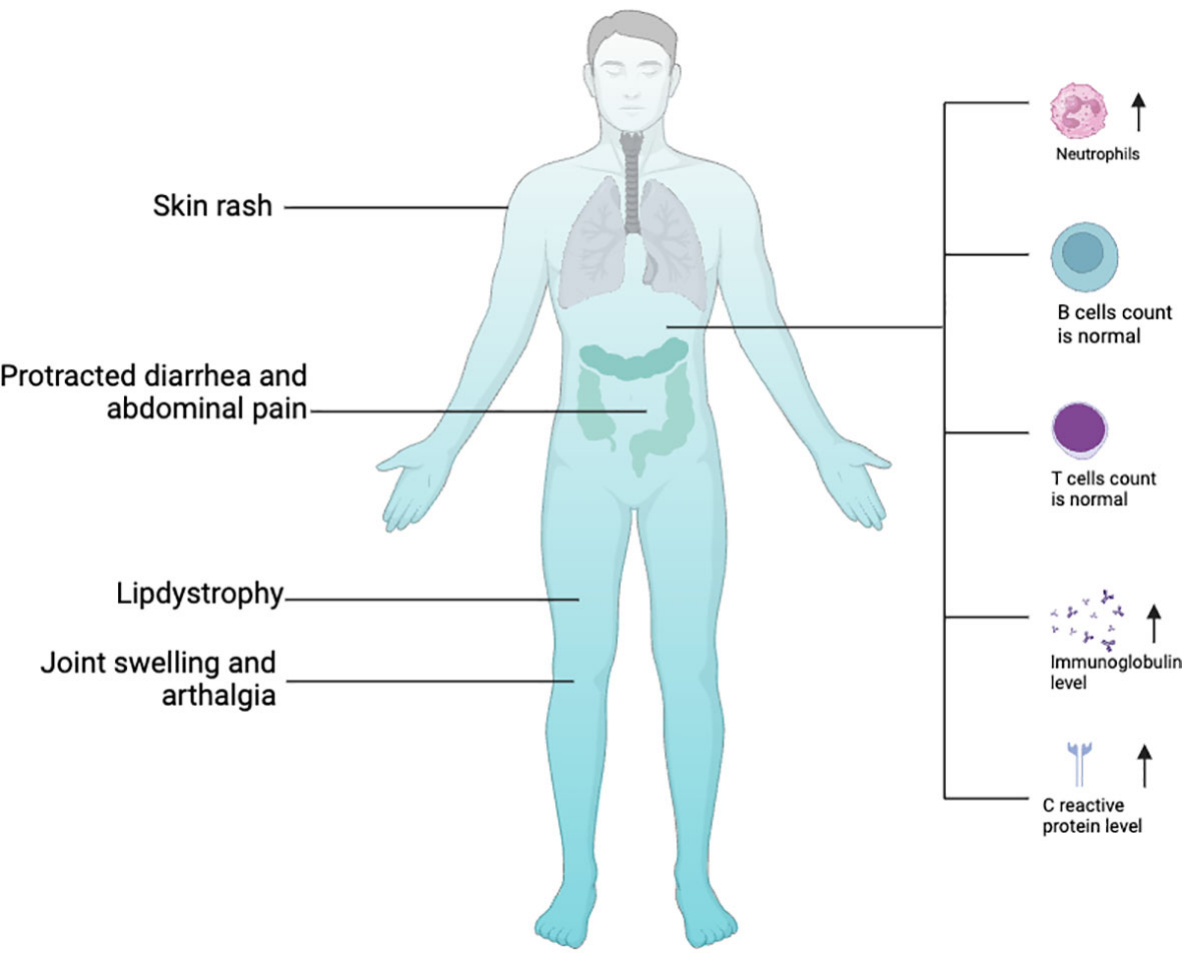
Clinical features of *OTULIN* with biallelic hypomorphic mutations. Image created with Biorender.com.

In 2016, two groups respectively identified a surplus of linear ubiquitin linkages in humans due to homozygous variants in OTULIN that result in an autoinflammatory disease: OTULIN‐related autoinflammatory syndrome (ORAS, also known as otulipenia) (30, 31). According to the 2022 Update of the IUIS Phenotypical Classification, OTULIN deficiency is classified among auto-immunodeficiency disorders of sterile inflammation, predominant at skin and joint (41). To date, 10 patients with ORAS from seven families have been identified (29–31, 40, 42, 43). The clinical landscapes of humans with biallelic hypomorphic mutations of OTULIN deficiency are complex and multifaceted (**Figure 2**) and dominated by premature birth at gestation from 28 + 6 weeks to 36 weeks, recurrent episodes of neonatal-onset fever, and rashes. Rashes showed pustules, scarring, or erythema with painful skin nodules. Skin biopsy revealed a predominantly septal panniculitis and neutrophilic dermatosis with vasculitis of small and medium-sized blood vessels. Other characteristic manifestations included protracted diarrhea, progressive lipodystrophy, joint swelling, arthralgia, failure to thrive, and spontaneous and progressive steatotic liver disease (30, 31, 44, 45). During the repeated episodes of systemic inflammation, patients present with elevated white blood cell count, serum C-reactive protein level, immunoglobulin levels, and serum autoantibodies without clear evidence for infections. Intriguingly, the raised white blood cell count comprises predominantly neutrophilia but normal lymphocyte subsets (30, 31). Consistent with this result, loss of OTULIN in T cells and B cells generated healthy mice with no overt inflammatory phenotypes, as assessed by blood cell, cytokine, and serum IgG analysis (30), whereas deletion of OTULIN in myeloid cells resulted in a strong inflammatory phenotype (30).

Using genetic testing, Mediterranean fever, chronic infantile neurological cutaneous articular syndrome, tumor necrosis factor-receptor associated periodic syndrome, and hyper IgD syndrome are excluded as the causes of the symptoms (Experimental Models and Subject Details). Four of the identified homozygous variants in the *OTULIN* gene, namely, p.Leu272Pro, p.Tyr244Cys, p.Gly174Aspfs*2, and p.Gly281Arg, are located in the OTU domain and confer predisposition of OTULIN stability, catalytic activity, and/or capability of binding to M1 ubiquitin (29–31), whereas the homozygous variant of c.864 + 2 T > C in *OTULIN* alters a donor splicing site and thus generates a truncated and unstable protein (40) (**Figure 1**). Numerous literatures had reported that disruption of LUBAC components lead to immunodeficient disorders and auto-inflammatory disorders. Loss-of-function mutations in HOIP or HOIL-1 are known to cause a severe multiorgan autoinflammatory disease in humans (14, 46) and lead to embryonic lethality in mice (11, 12). In line with the role of OTULIN in counteracting LUBAC, OTULIN-deficient skin fibroblasts from patients with ORAS showed reduced TNF-induced NF-κB and MAPK activation but enhanced sensitization to TNF-induced cell death relative to healthy controls (29). Intriguingly, the functional consequences of OTULIN deficiency in ORAS are cell type-specific (29, 31, 39). OTULIN deficiency in myeloid cells has been shown to result in increased NF-κB signaling and MAPK activation and cell death responses *in vitro* (29, 47). Patient-derived fibroblasts with OTULIN deficiency show a diminished response to TNF (29). Currently, there is limited evidence regarding OTULIN’s role in T cells. This may disrupt the homeostasis of T cells, leading to autoimmune diseases. However, OTULIN deficiency in epithelial cells disrupts intestinal barrier integrity and exacerbates intestinal inflammation, contributing to the pathogenesis of inflammatory bowel disease in ORAS (48). Nevertheless, the molecular mechanism of these cell type-dependent differences remains elusive.

Patients with biallelic hypomorphic variants in the *OTULIN* gene mainly present with an early-onset ORAS, whereas one Greek patient with two compound heterozygous variants in *OTULIN* was associated with fulminate atypical late-onset ORAS (29–31, 39). This has led to an expansion of the clinical spectrum of ORAS patients (39). This Greek patient, a 7-year-old boy, demonstrated age-appropriate psychomotor development and obesity features. He experienced a severe autoinflammatory episode characterized by multiorgan sterile abscess formation. Initially presenting with abdominal pain, subfebrile fever, leukocytosis, neutrophilia, and significantly elevated levels of C-reactive protein, the patient subsequently developed spiking fevers, inflammatory lesions on the wrist and ankle, and abscesses in the left lower pulmonary lobe, left axilla, and spleen. Inflammatory parameters continued to rise during the course of his illness. However, treatment with broad-spectrum antibiotics had not improved the patient’s condition, biopsies and smear remained sterile, and histopathological analysis of the skin revealed massive inflammatory infiltrates, predominantly consisting of granulocytes, monocytes, and macrophages. Lung and spleen showed signs of inflammation and necrosis. All blood culture, stool culture, and tracheal fluids remained sterile. On day 14 of in-patient stay, an autoinflammatory syndrome was suspected, and treatment with corticosteroids was started, which resulted in a decline of body temperature and CRP levels and in marked improvement of the patient’s condition. Two months following admission, the patient was discharged in good condition. Using a combination of whole exome sequencing and targeted Sanger sequencing, a compound heterozygous missense variant in exon 3 (c.258G>A; p.M86I) and exon 5 (c.500G>C; p.W167S) of the *OTULIN* gene was revealed (39). The patient inherited the p.M86I variant from his father, whereas his mother is a heterozygous carrier of the p.W167S mutation. The patient is the second child born to non-consanguineous parents. Both his parents and siblings are clinically well. In-depth biochemical analysis revealed that binding of OTULIN to linear ubiquitin is compromised by both variants; however, protein stability and catalytic activity are most affected by the *OTULIN* variant p.W167S (39).

Another possible clinical feature of OTULIN deficiency is embryonic lethal. Mice carrying loss-of-function homozygous variants W96R and D336E in *Otulin* resulted in abnormal cranial vasculature and die embryonically after embryonic day E12.5 (18). Genetic loss of *Otulin* in mice or expression of a knock-in C129A variant leads to embryonic lethality at E10.5 (30, 35), with the C129A-expression mice showing extensive cell death in the yolk sac and placenta (35). Tamoxifen-induced adult CreERT2-OtulinLacZ/flox mice become moribund within a day (30). Deficiency in functional OTULIN protein by these mutations suggest a potential role of the LUBAC/OTULIN axis in developmental Wnt signaling (18, 49), though exactly molecular mechanisms are unclear.

### 3.2 OTULIN haploinsufficiency underlies an immunodeficient disorder conferring predisposition of life-threatening staphylococcal disease

Autosomal recessive *OTULIN* mutations cause ORAS, manifesting early in life with autoinflammation without clear evidence of infections, due to the defective downregulation of NF-κB-dependent inflammatory signaling. Recently, a group of patients carrying heterozygous variants of *OTULIN* were identified (named immunodeficiency 107 by MedGen) (38, 43, 50). Clinical manifestations presented, to some extent, with phenotypic heterogeneity. Six carriers were apparently healthy, one-third of patients expressed relatively milder respiratory infections, and the other one-third expressed the severe phenotypes including necrotizing pneumonia, necrotizing cellulitis, recurrent furunculosis, abdominal sepsis, spontaneous abscess, and sepsis shock, thus indicating that rare heterozygous mutations of *OTULIN* confer predisposition to severe necrosis, typically, but not exclusively, after infection with *Staphylococcus aureus*. It should be mentioned that skin and/or lung are consistently affected in all cases. The age of clinical onset is variable, and the earliest age of clinical onset in this cohort is 1.5 years (38, 43). The penetrance is estimated at 90%.

In comparison with biallelic *OTULIN* deficiency, OTULIN haploinsufficiency causes an accumulation of linear ubiquitin in dermal fibroblasts, but TNF-receptor NF-κB signaling remains intact. Routine immunological tests including assessments of leukocyte differentiation and oxidative burst capacity revealed no explanatory defects in patients with OTULIN haploinsufficiency. Subtle immunological testing for peripheral blood mononuclear cells (PBMCs) by mass cytometry and RNA sequencing showed no differences in the abundance of leukocyte subsets or in the expression of lineage markers. The development of myeloid and lymphoid subsets in patients with OTULIN haploinsufficiency was normal. Both at baseline and after stimulation, the PBMCs of the patients had a capacity to secrete various cytokines, including TNF and IL-1β, similar to that of PBMCs from healthy controls (38). These observations indicate that human OTULIN haploinsufficiency conferring susceptibility to infections is driven by a defect of cell-intrinsic immunity in fibroblasts.

## 4 Current management and future perspectives

Given the broad spectrum of clinical manifestations, the diagnosis of OTULIN deficiency can be challenging. A strong clinical suspicion must arise when clinical features, such as neonatal onset of recurrent fever, erythematous rash with painful nodules, and painful joints, are presented. Because of the auto-inflammatory nature of ORAS, anti-inflammatory drugs currently represent an effective treatment. However, treatment of affected patients with prednisolone (general cortico-steroidal immunosuppressant), azathioprine, methotrexate (anti-proliferative immunosuppressive drugs), and Anakinra (recombinant IL-1R-antatonist) had little or no effect on clinical symptoms (30, 31). By contrast, TNF-blocking agents infliximab or etanercept drastically reduced the symptoms, and serum levels of CRP as well as WBC and neutrophil counts in blood returned to normal ranges, suggesting a TNF-mediated pathogenesis in the patients (30, 31). Hematopoietic stem cell transplantation (HSCT) has been treated in one patient who developed severe inflammatory symptoms after birth, including fever and panniculitis (29). At 7 months of age, this patient had fever, diarrhea, panniculitis, cachexia, and cataracts. She underwent an HSCT at 17 months of age, and her symptoms resolved for 9 months, at which point she relapsed with fever and panniculitis. It was found that she had a decrease in bone marrow chimerism and regrowth of native hematopoietic cells. At 29 months of age, she was treated with Etanercept and had resolution of symptoms (29).

Further research needs to explore effective treatments other than anti-inflammatory drugs. Gene editing technology, such as the CRISPR/Cas9 system, represents one of the most curative approaches, in the wake of a successful outcome in the treatment of other hereditary diseases. In addition, the use of homology-direct repair and somatic genetic rescue as a correction mechanism may be attempted.

## 5 Conclusions

Germline mutations of *OTULIN* cause various complex clinical phenotypes, including autoinflammatory disorders and immunodeficient disorders. Despite the emergent findings, the exact pathogenetic details underlying these genetic variants are elusive. Uncovering these subtle molecular mechanisms will help develop more efficient and targeted treatments for patients.

## Author contributions

CL: Funding acquisition, Supervision, Visualization, Writing – review & editing, Writing – original draft. BD: Investigation, Writing – original draft, Data curation. WP: Writing – original draft. GJ: Investigation, Data curation, Writing – original draft.
